# The Cross-Country Comparison Model for Labor Participation (CCC Model for LP) of Persons with Chronic Diseases

**DOI:** 10.1007/s10926-022-10041-y

**Published:** 2022-06-20

**Authors:** Angelique de Rijk, Karina Carrasco-Negüe, Inge Houkes

**Affiliations:** 1 Department of Social Medicine, Care and Public Health Research Institute CAPHRI, Faculty of Health, Medicine and Life Sciences, Maastricht University, P.O. Box 616, Maastricht, 6200 MD the Netherlands; 2 Department of Obstetrics, School of Medicine, Pontificia Universidad Católica de Chile, Santiago, Chile

**Keywords:** Cross-cultural comparison, Chronic Disease, Employment, Models (Theoretical), Social Theory

## Abstract

*Purpose* To design a model based on the three pillars of new institutional theory (NIT), that facilitates cross-country comparison of labor participation (LP) of people with chronic diseases. This model should support getting a comprehensive overview of factors representing country differences, understanding these differences and should support estimating cross-country transferability of policies and interventions in the context of Work Disability Prevention. *Methods* Based on NIT, a draft model was designed by means of (1) a literature review of empirical studies; (2) theoretical books and articles; (3) a focus group with six expert researchers. This draft model was (4) adapted in the context of academic education. Literature was searched on Web of Science and EBSCO host. Feedback on (use of) the model was received from the focus group, four different academic courses at 28 occasions and two international conferences. *Results* The cross-country comparison model for labor participation (CCC model for LP) of persons with chronic diseases is proposed consisting of five factors: (1) Legislation; (2) Norms & values in practice; (3) Culture; (4) Organization of WDP in practice; (5) Labor market characteristics. Within these factors and based on (in)direct empirical evidence, subfactors are distinguished. The feedback received led to renaming (sub) factors, improved visual representation and a tool for estimating transferability. *Conclusions* The CCC model for LP of persons with chronic diseases allows for a comprehensive understanding of country differences and cross-country transferability of policies and interventions. The CCC model can be used for other populations when population-specific subfactors are included.

## Introduction

The employment rate of people with chronic diseases is lower than that of the general population [1, 2] although the exact number varies across countries [1, 3, 4]. These cross-country differences in labor participation (LP) are not yet fully understood. Comparing countries could help to draw lessons from one another [5] and increase the employment rate of people with a chronic disease, an important aim of Work Disability Prevention (WDP) [6]. Policy researchers making cross-country comparisons would strongly benefit from a model [7]. Further, there is a need for assessing the transferability of legislation, public policy and interventions from one country to another, accounting for national differences [8, 9]. Finally and more generally, in a globalizing research community, there is a stronger need for more systematic comparison of results from different countries [8–10]. There are thus practical, theoretical and empirical reasons for the development of a model to perform cross-country comparisons regarding LP of persons with chronic diseases in the context of WDP. To the best of our knowledge, such a model is not yet available.

This model should represent societal context factors, e.g. type of governance and culture [3, 11, 12]. Recently, Nielsen and colleagues [13] developed the IGLOO (Individual, Group, Leader, Organization and Overarching/social context) model for the resources that promote return to work of persons with common mental health disorders. They point to the cascading effect that both national policies and negative portraits of persons with disabilities in media can have on organisational policies and practices, and criticize the lack of cross-country comparisons [13]. Regarding cross-country comparisons of LP of people with chronic conditions, a few studies compared welfare regimes, sickness absence systems, labor market and macro-level policies, and interventions for people with chronic diseases and/or disabilities between countries [3, 10–12, 14]. These studies focused on only a few and apparently arbitrary aspects of the societal context without strong theoretical underpinnings.

### New Institutional Theory (NIT) as a Theoretical Basis

We propose the New Institutional Theory (NIT), a sociological theory, as a starting point for designing a model for cross-country comparison of LP of people with chronic diseases. Institutions are the rules and norms [15] that give society its order and structure. NIT combines different institutional approaches and assumes that institutions shape behaviour by both constraining and empowering activities such as LP in our case [16]. We will use the three types of institutions distinguished by Scott [16] as basic factors for our model. These are the regulative, normative and cultural-cognitive institutions renamed for the purpose of our model as legislation, norms and values, and culture respectively. The three types of institutions constitute a continuum that moves from the conscious (e.g. legal regulations) to the unconscious (e.g. taken for granted patterns) [16]. Institutions interrelate: external constraints (e.g. legal regulations) as well as internalized models (e.g. taken for granted patterns) may produce regular patterns of social behaviour. The patterns might become stronger and more stable over time as the institutions get internalized [17].Kümpers et al. [18], van Raak et al.[19] and Den Dulk et al. [20] have successfully used NIT as theoretical basis for understanding cross-country differences in health policy for elderly, re-integration into work after sickness absence, and the adoption of workplace work-life polices in Europe respectively. The basic factors are defined below.

1. Legislation. Regulative institutions constrain and regularize behaviour. The major types are governmental legislation and policies. Regulative institutions are the rules combined with their enforcement mechanisms such as monitoring and sanctioning [16, 21], which are formally constructed and written down [22].
2. Norms and values. Normative institutions are the normative systems that comprise values and norms in a certain group, such as a professional group. Values represent the views of the preferred or the desirable. Norms specify ‘how things should be done’ and define the legitimate means to pursue valued ends. Norms and values are trained in (professional) education. Normative institutions impose constraints and at the same time empower and enable behaviours [16, 23], e.g. depending on the content, norms of health care professionals regarding paid work could encourage or discourage persons with chronic diseases to participate in paid work.
3. Culture. Cultural-cognitive institutions involve shared meanings, common modes of interpretation and shared understanding of experiences within a certain culture. These common understandings allow daily activities to become routinized and taken for granted [24]. A pattern that is taken for granted may be perceived as an external constraint, even though the individual is not totally conscious of this pattern [25]. This type of pattern is reproduced daily and legitimated by the social environment in a more indirect way than norms and values. Although there is some discussion on the topic, cultural-cognitive institutions can be regarded as ‘culture’ [18, 22]. Cultural-cognitive institutions are able to both constrain and enable behaviours [26].
4. How practice is organized. In order to study cross-country differences, it was thought important to add this fourth factor. Over time, institutionalized institutions might lead to the development of social structures [16, 27, 28], social entities that have a certain order, organization or hierarchy and a degree of stability in time and space [29]. Legislation–a regulative institution–often introduces certain professionals or organisations. Also, the exertion of legislation on work disability goes together with the availability of a social insurance institute with insurance physicians. The combination of institutions with their (formal) organizations is called ‘configuration’ [18]. Institutions might prevail over organisations, though. In a study on how, in practice, career success was defined in multinationals, country differences were large and could be explained by institutional differences [30].

### Designing a Model for LP of Persons with a Chronic Disease: Adding Subfactors

Legislation, norms and values, culture and ‘how practice is organized’ are thus regarded as the four building blocks of the model. However, these are broad and general factors, and we propose to identify subfactors that represent the background of cross-country differences in the LP of persons with a chronic disease, to allow for a comprehensive understanding.

### Contributing to Transferability Studies

We further argue that a model built on NIT will also contribute to studying cross-country comparison but also to studying transferability of legislation, policy and interventions, and will add to existing checklists for assessing transferability in economic evaluation studies [8, 9]. Transferability relates to the degree to which the institutional context of the initial legislation, policy or intervention is comparable with the new institutional context. Higher transferability implies better implementation, while worse transferability implies that the legislation, policy or intervention first needs to be adapted to the country’s context [30]. The above-mentioned checklists are not theory-based and neglect institutions such as cultural factors that might be important barriers to implementation. For example, in a study at the beginning of this century, Dutch sickness absence legislation was badly implemented compared to Belgium because the Dutch lacked a culture of routine use of the new legislation [25].

### Being Useful for Academic Teaching

Finally, cross-country comparisons and assessing transferability of legislation, a policy or intervention to another country are important topics for researchers and policymakers, but also for the academic teaching curricula. Therefore, our model needs to be instructional for its users. Following a participatory design perspective, academic education and conferences can be used to improve a model [31].

The objective of this paper is thus to design a model for making cross-country comparisons of LP of persons with chronic diseases, having NIT as theoretical basis, and further using empirical literature and expert knowledge. Specific aims are that the model should be helpful in:

1. Getting a comprehensive overview of (sub)factors representing institutional differences between countries
2. Understanding the background of such differences in LP of persons with chronic diseases
3. Estimating the degree of transferability of legislation, policy or interventions from one country to another country
4. Teaching on cross-country comparisons of LP of persons with chronic diseases in academia.

## Methods

Starting from institutional theory and the four factors presented in the introduction, we designed a model for cross-country comparison of the LP of persons with a chronic disease using four methods: (1) a literature review of empirical studies; (2) consultation of theoretical books and articles, (3) a focus group with experts and (4) using and adapting the model in the context of academic education and international conferences during 6 years. On the basis of (1) and (2), a draft model was developed, which was adapted after (3) and (4).

The initial literature review was performed in 2014, in the Web of Sciences and EBSCO host (including Business Source Premier, CINAHL, ERIC, SocINDEX and MEDLINE databases), topic specific electronic journals (International Journal of Comparative Labour and Industrial Relations, LABOUR Review of Labour Economics and Industrial Relations, E-journal of International and Comparative labour studies, Chronic Illness, Labour Capital and Society, Socio-economic Review, and International Journal of Disability Management). We searched for articles published between 2004 and 2014. First the search terms in the three first columns of Table 1 were used, and on the basis of the selected articles, search terms were added (fourth and fifth columns of Table 1) to search more specific studies. The reference lists of the retrieved articles were also searched.

**Table 1:** Key words for literature review

Population	Phenomenon	Comparison	Social context	
Chronic disease(s)	Labour participation	Comparison	Welfare state	Norms
Chronic illness	Labour	Cross-country	Welfare state regimes	Values
Illness	Work	Countries	Comparative welfare state	Sick role
Sickness	Employment	International differences	Health care system	Work values
Chronically ill		Cross-cultural differences	Health care access	Culture
			Health service accessibility	Context and social meaning

Inclusion criteria were that the articles should address country comparison, LP and contextual social factors (institutions and labor market characteristics, although they might have been phrased differently). These first two steps were explorative and iterative and resulted in a selection of 93 articles selected by title. Then, the abstracts were read, which resulted in 30 relevant articles that were all read and analysed. The articles were discussed by two of the authors (AdR and KCN) and from each relevant article, the information about the relationship between the four factors, and with LP of people with chronic conditions was extracted and summarized. Upon agreement between the two first authors, the subfactors mentioned in the articles, were categorized according to the four factors based on NIT introduced in the introduction: legislation, norms and values, culture, and how practice is organized. Within these categories, a distinction was made between theoretical and empirical literature and between literature focusing on labor participation and/or persons with a chronic disease, and general literature.

Second, books on NIT, social structures, culture, cross-country comparisons, comparative politics and welfare states were consulted by KCN. The information was summarized, and then whether and how this theoretical information supplemented the empirical studies or not was discussed between the two first authors. After this process, a draft model was made.

Third, the draft model was discussed in a focus group of researchers in the field of LP and/or international comparative research with the aim to refine or adding factors in the model, based on expert knowledge. Five researchers participated in this focus group; an additional individual meeting was held with a sixth researcher who was unable to attend the focus group. The meetings were recorded, and written notes were taken. Under the Dutch legislation, ethics approval by an ethical committee for a focus group of researchers with the aim to exchange thoughts and knowledge –the regular professional activity of scientists–and not exchanging personal experiences, was not mandatory. The authors of this manuscript followed the Helsinki Declaration though, including anonymous and confidential treatment of recordings and notes.

Fourth, the model that resulted from step 3 was used in 28 occasions (between 2014 and 2021) in four different academic courses for international groups of students at Maastricht University, the Netherlands: second year Health Sciences; Master Work, Health & Career; Master Medicine, and two occasions of a postgraduate international course for researchers and practitioners in the field of WDP organized by the Nordic Institute for Advanced Training in Occupational Health (NIVA). Groups varied between 6 and 40 students. Students were instructed about the model in an interactive lecture and received an assignment for a paper (Health Sciences) or presentation (other courses) to estimate the transferability of a work and health policy or intervention from one country to another (Health Sciences), or to compare the LP rate of persons with chronic diseases between two countries (other courses). After each course, the students’ assignments and regular feedback from students and tutors were checked for improvements in the clarity and feasibility of the model. Further, the model was presented at two international conferences: (1) the preconference “Extending working life beyond the age of 50; challenges in cross-national research” of the European Public Health conference (Vienna, 2016), where it was applied to the LP of older workers (with and without chronic diseases); (2) as a Key Note at the European Medical Association for Social Security (EUMASS) conference (Maastricht, 2018). Points from the discussions and the oral feedback from the audience (researchers and practitioners) were collected, additional literature to confirm or reject the comments was searched for, all was processed into an adapted model and tested in the next academic session by the first author. In December 2021, the literature was checked for another cross-country comparison model for LP of persons with a chronic disease. The final model was agreed upon by all authors.

## Results

**Fig. 1:**
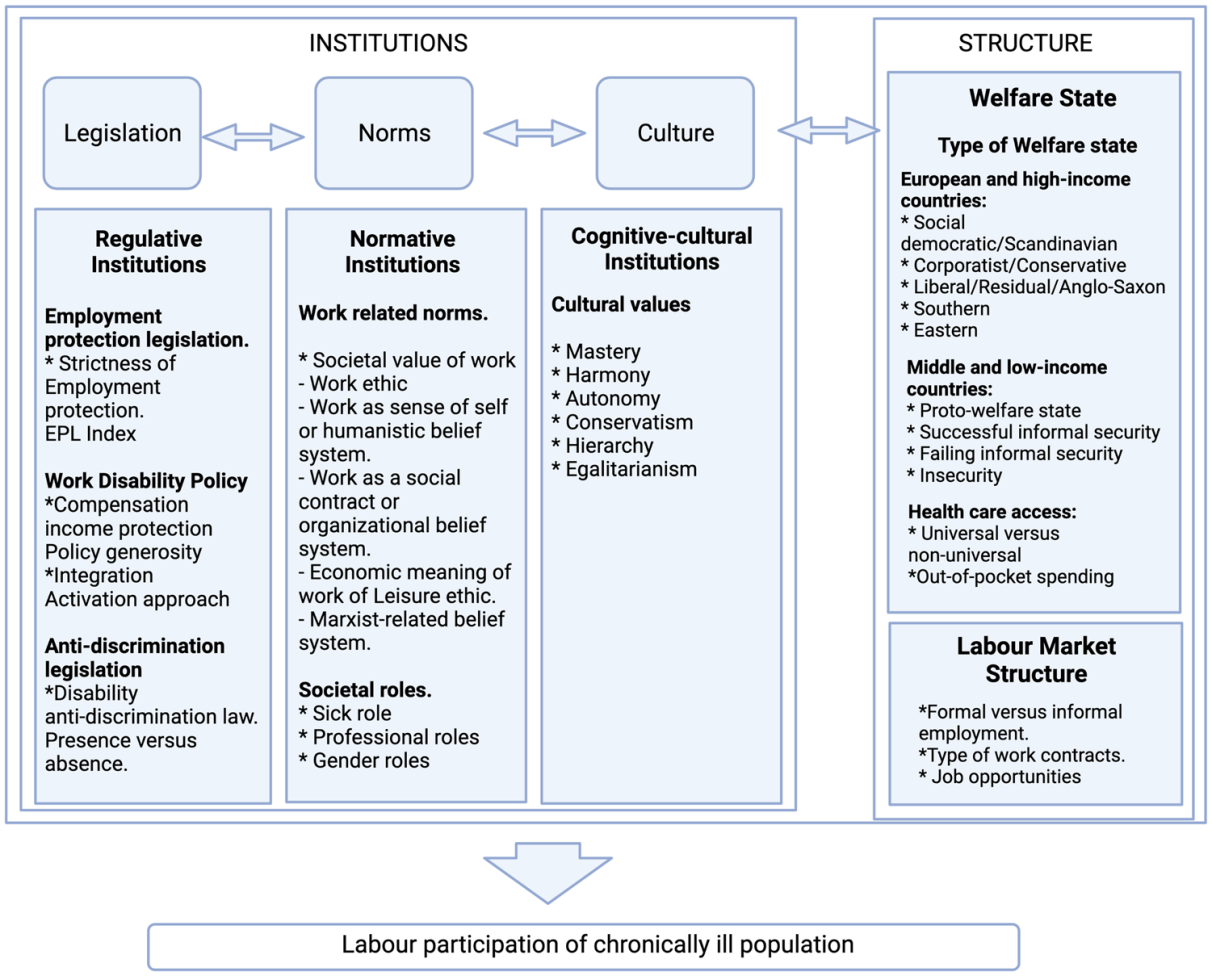
Draft model

The literature review and search for theoretical literature (methods 1 and 2) lead to five main factors: the three institutions, the organization of work disability prevention and, added on the basis of literature, the country’s labor market characteristics. Within these five factors, specific subfactors were identified. Based on these findings we elaborated a draft model (Fig. 1.) For most subfactors (some) empirical evidence on the direction of the relationship between the subfactor and the labor participation rate of persons with chronic diseases was found; a few relations could only be based on theoretical reasoning. The majority of articles was from high income countries, with a bias towards European countries.

During the focus group, (method 3), subfactors were moved to another main factor and subfactors were more precisely defined. For example, type of welfare state was recognized as part of legislation. Additionally, experts improved the definition of health care access which also includes type of payment and quality. Finally, and after a discussion on the implications of a culture of collectivism, they recommended to use the concept of “Culture types” instead of “Cultural values.” Regarding labor market, they added labor market flexibility, the type of employment besides working part-time, being self-employed or not and quality of work.

**Fig. 2:**
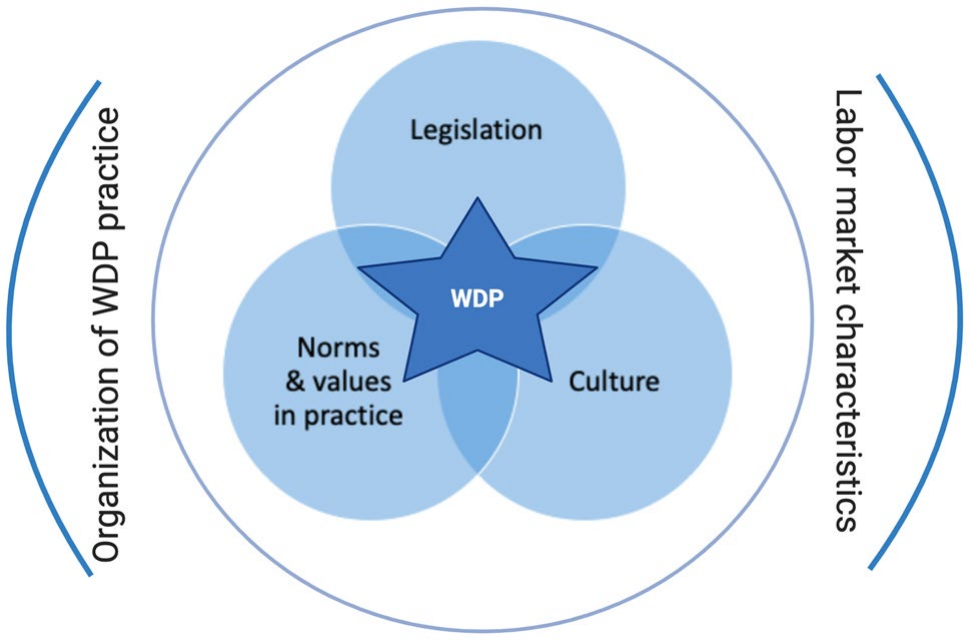
Generic CCC model for LP

**Fig. 3:**
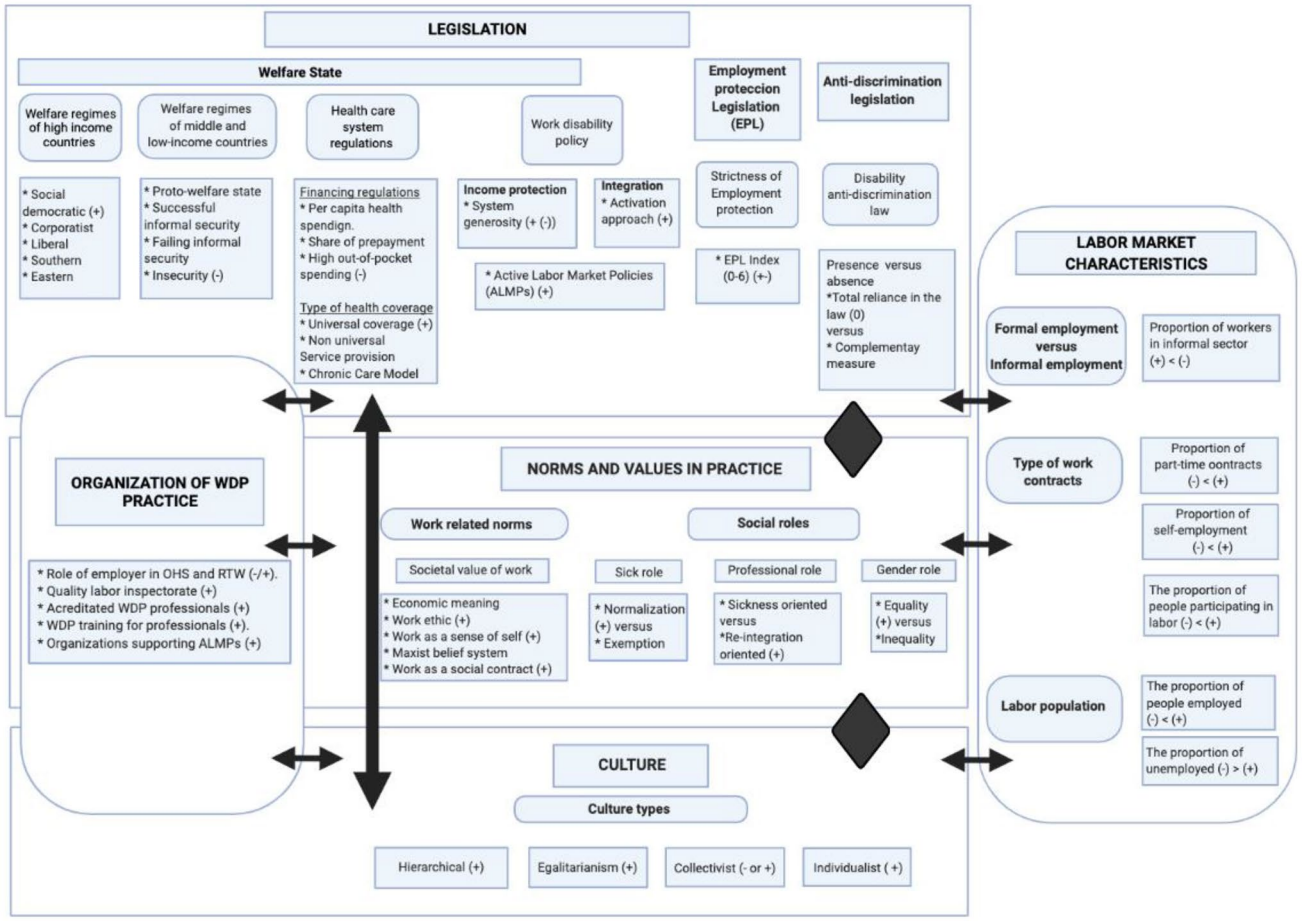
Comprehensive CCC model for LP of persons with a chronic disease, including subfactors

The teaching and conference settings (method 4) mainly reflected unclarities about NIT and–as a consequence—the naming of the (sub) factors, their definitions, the visual representation and how to assess transferability. The students and conference visitors understood how to use the model for comparing countries, but struggled with the use of the model for estimating transferability of WDP laws, policies and interventions. How can the model be used to get a global impression of whether for example, a new return-to-work policy presented at a conference, could successfully be transferred to one’s country? Therefore, several adjustments have been made in response to the focus group, students and conference participants. First, we decided to replace the original NIT terminology with more common sense names for the factors. Second, two visual representations of the model were developed: (1) an easy-to–understand generic model including the five main factors; and (2) a comprehensive model including all subfactors. Several drafts were developed and tested in subsequent student groups before the final visual representations were composed (see Figs. 2 and 3). Students appeared to be able to find information on subfactors by document study, literature review and interviewing stakeholders on norms, values and routines regarding the country’s WDP policy. Third, a simple tool to quickly get a first impression of transferability was developed along with the model, and its use tested in several student groups (this tool will be presented at the end of this section). Fourth, new empirical evidence was added in response to the focus group advice and along with keeping the lectures up-to-date and responding to student evaluations. Ultimately, subsequent student groups did not complain anymore about understanding the model and saturation was achieved in perceiving the model as instructional for their assignments. The generic five-factor representation of the final cross-country comparison model for LP (CCC model for LP) of persons with chronic diseases is presented in Fig. 2.

Below, the five factors and their subfactors, based on the literature review, are explained (see also Fig. 3 for the comprehensive representation of the CCC model for LP, including direction (+ or − ) of the relationship between subfactor and LP, based on empirical evidence).

### Legislation

Legislation refers to the type of welfare state (including welfare regimes of high and of middle and low-income countries, health care system regulations and work disability policy), employment protection legislation (ELP), and anti-discrimination legislation.

#### Type of Welfare State

##### Welfare Regimes in High Versus Middle and Low-Income Countries

The welfare regime is the role of the state in relation to education, health, housing, poor relief and other social services [32] and can be categorized regarding the division of responsibilities between the interdependent sources of social welfare and security: government, civil society and the market [33]. Generally, welfare states shape how vulnerable citizens are treated [33] and employment structures [34], and both affect labor market outcomes [32, 34].

There are different theories for the welfare regimes of high and of middle and low-income countries. Regarding high-income countries, Esping-Andersen [34] and Bambra and Eikemo [35] distinguish three types of European welfare state regimes: Liberal (Residual or Anglo-Saxon), Corporatist-Conservative (Conservative or Bismarckian) and Social-Democratic (Scandinavian). More recently the Southern welfare state and Eastern welfare state have been added [35].

For middle and low-income countries, such as Chile, Brazil, and Poland, the classification of Sharkh and Gough [36] distinguishes four types. First, Proto-welfare state regimes are characterized by a relatively important commitment from the state to provide welfare and relatively effective service delivery, reflected in moderate social security. Second, Successful Informal Security regimes combine a relatively good welfare outcome and service delivery with a low level of social spending by the state. Failing Informal Security regimes include two sub-clusters: (1) high illiteracy, low levels of women enrolment in secondary education, but more extensive democracy and income equality; (2) high literacy, and increased expenditure on and delivery of public social policies. Fourthly, Insecurity Regimes are characterized by low and decreasing life expectancy, low levels of public spending and social services and the absence of informal security mechanisms [36]. In these welfare state regimes, LP might still be relatively high, as no income protection will exist.

*Welfare State and LP of Persons with Chronic Diseases* There is widespread evidence from cross-country comparisons that the type of welfare state affects LP of persons with chronic diseases [37, 38]. In a study comparing 26 European countries, it was demonstrated that people with chronic diseases had the lowest unemployment rate in the Scandinavian welfare regimes and the highest in the Anglo-Saxon and Eastern welfare regimes [38]. This is in line with the study of Anema et al. [12] who found that the high-income countries which have more generous welfare regimes produce more sustainable LP, explained by a stronger focus on return-to-work policies [12] (see below as well).

##### Health Care System Regulations

Health care is the largest area of welfare state service provision [39] and legislation can affect people with chronic diseases in particular [40, 41]. Health care systems are categorized according to how they fulfil their four basic functions: financing, service provision, resource generation and stewardship [42]. Financing is the process by which revenues are collected, accumulated and relocated to specific health actions, and represented by two subfactors:

- Total per capita health spending [2].
- The share of prepayment for health expenditures, in contrast to out-of-pocket spending and reflecting the degree of protection against major health costs s [43]. Service provision refers to the combination of inputs to deliver health actions and interventions [44, 45] and represented by two not mutually exclusive subfactors:
- Coverage for health care: the part of the population that is guaranteed of receiving a core set of health care [46].
- The extent to which the Chronic Care Model (CCM) is adopted. The CCM includes several components, and the following subfactors have shown to improve quality of care and clinical outcomes among chronically ill [47, 48]:
- Self-management support: the process of providing information and support that allow the individual affected by a chronic illness and its family to care better for his or her health condition.
- Delivery system design: must favour productive and satisfying interactions, around chronic illness, between patients and health care teams.
- Decision support by health care providers. At the same time, health care providers must have access to the necessary expertise to care for patients with chronic conditions.
- Clinical information systems that include disease registry or database that allows access to all information about the care process and outcomes of care in the patients.

*Health Care System and LP of Persons with Chronic Diseases* With respect to health care financing function, the share of prepayment varies by country and improves access to and use of health care, which might be advantageous for persons with chronic diseases and their LP. Among OECD countries, it has been shown that the out-of-pocket medical spending (excluding long-term care health expenditure) ranges from 4.6% (Chile, México and Korea) to 1.5% (United Kingdom, Turkey and Netherlands) of the share of final household consumption (OECD average is 2.9%) [46]. The United States (US) had the highest out-of-pocket spending as compared to Australia, Canada, France, Germany, the Netherlands, New Zealand, the United Kingdom (UK) [49]. Relatively high out-of-pocket spending creates a barrier to health care access and use [46], which is clearly confirmed for the U.S. [33, 50].

Coverage for health care can also affect access to health care. When health care coverage is not universal (e.g., in the US), more citizens have difficulties accessing health care [46, 51]. Conversely, in the UK, with universal health care coverage, adults are less likely to report access problems and disparities in quality of care are small or absent [33, 34]. A cross-country comparison that included persons of fifty years and older from Sweden, Germany, the Czech Republic, the UK and the US showed that US had the poorest level of physical health and UK had the highest level. In all countries a positive association between household wealth and health was found, with a remarkably strong association in US explained by the lack of universal coverage and expensive private insurance system [52].

As regards health care provision, evidence suggests that people with chronic diseases who receive effective treatments, self-management support and regular follow-up have better health outcomes. This might affect their LP positively [33, 53].

##### Work Disability Policy

Chronic diseases often relate to (partial) work disability, thus work disability policy is an important contextual factor for LP of persons with chronic diseases [14]. Work disability policy has two main dimensions: income compensation or protection, and integration into work [1, 14, 54]. The first dimension relates to the characteristics of the disability benefits scheme, and the integration dimension corresponds to all measures focused on employment and rehabilitation [14]. Countries differ in the emphasis that they put on one of these two dimensions [1, 11, 12, 14]. The OECD (1) developed two “disability policy indicators” to facilitate cross-country comparison of work disability policy: the compensation dimension and integration dimension. A higher score on the compensation dimension indicates greater system generosity and a higher score in the integration dimension indicates a more active approach towards participation in work. The latter is also referred to as Active Labor Market Policies (ALMPs) [37].

*Work Disability Policy and LP of Persons with Chronic Diseases* Large differences are found between countries regarding their work disability policy [1, 11, 12, 55], and there is widespread evidence that this affects LP among persons with chronic diseases [12, 56]. In a comparison of 26 European countries, welfare generosity and spending on ALMPs was associated with higher LP among persons with chronic diseases. Iceland had the welfare state with the highest level of generosity and the lowest unemployment rate, and also the other Nordic countries with social democratic welfare systems have such positive results. One explanation might be that in circumstances of less insecurity, vulnerable citizens have higher self-esteem and ability to cope [37]. Anema et al. [12] found also that the generous social-democratic welfare state was better for returning to work as it allows persons with chronic diseases in the benefit system when their health condition detoriates. Other studies confirm the positive effects of state ALMPs expenditure on the LP of vulnerable groups, as this allows them to bounce back [1, 56, 57]. Some studies show that accessibility and generosity of disability benefits reduce the employment rate in disabled persons, but these studies are of weak quality tend to neglect the adverse effects of poverty [57]. Overall, the evidence for system generosity increasing the LP of persons with chronic diseases is more convincing.

#### Employment Protection Legislation

Employment protection legislation (EPL) comprises the regulative rules of hiring and firing workers, in order to safeguard workers against job loss and the society against the costs of job loss [40]. The strictness of employment protection clearly varies across countries [40, 58]. The OECD [58] developed the EPL index, that includes eighteen basic items classified into three groups: (1) employment protection of regular workers against individual dismissal; (2) specific requirements for collective dismissal; and (3) regulation of temporary forms of employment. The EPL index ranges from 0 to 6 points score, with a higher score representing stricter regulation [39].

*Employment protection legislation and LP of persons with chronic diseases* The degree of labor market flexibility and de-regulation may have an impact on persons with chronic diseases [11], who have higher risk of unemployment [41]. EPL can be protective against dismissal. Reeves and colleagues [59] found that EPL had a protective effect against job loss among people with chronic conditions, especially women, except during periods of economic recessions. In a comparison between UK, Canada, Denmark, Norway and Sweden, these three latter countries showed the highest EPL index and had a better employment rate of persons with chronic diseases [56]. EPL does not affect unemployment but stringent EPL affects the entrance of young people into the labor market negatively [60]. Thus, too strict EPL could prevent persons with chronic diseases to be hired.

#### Disability Anti-Discrimination Legislation

Disability discrimination might occur when employers in recruitment processes use “disability” as exclusion criterion [61, 62]. Given the vulnerability of disabled people, including persons with chronic diseases, regarding discrimination at work, several countries have enacted disability anti-discrimination laws [63]. Since 1997 (Amsterdam Treaty) the European Union principle of non-discrimination in the labor market was extended to disability [64] for Europe.

*Anti-discrimination legislation and LP of persons with chronic diseases* A comparison of the effects of anti-discrimination legislation in the US and in Canada showed some differences. The Americans with Disabilities Act (ADA) in the US have made important improvements in the accessibility of disabled people to physical environments such as buildings, schools, transport and so on. However, in terms of access to employment ADA has shown to be ineffective, and courts do not help to accommodate people in the workplace. Canadian courts were more protective of disabled people against discrimination in the workplace than US courts. However, Canadian legislation has made no improvement in terms of physical environment accessibility for disabled people, and showed no impact on improving their LP rate [65]. Similarly, Bell and Heitmueller [66] found that the Disability Discrimination Act in the UK had no impact on LP among disabled people. The existence of anti-discrimination legislation correlates with higher levels of disability benefit recipients [1]. The failure of anti-discrimination legislation in raising the LP among persons with chronic diseases may be explained by its case-wise approach, and many workers with chronic diseases might lack the financial, energy and intellectual resources to go to court.

### Norms and Values in Practice

Particularly in meritocratic societies, work, action and having control are highly valued [32], and as such are strong normative institutions. In this social context, persons with chronic diseases might experience stigma [32, 67], frustration, guilt and suffering associated with the inability to accomplish expected roles such as being productive and able to work [67, 68].

#### Work-Related Norms and Values

Beliefs about work vary across societies [69]. Generally, having a paid job is a positively valued activity, and being unemployed has less social value than being employed [32, 70, 71], but particularly the function and meaning of work can vary across societies. Buchholz [72] distinguished five different belief systems about the nature of work [72]. According to the humanistic belief system, work is a way of personal development and fulfilment. Thus, personal growth and development on the job are more important than work outputs such as money. Marxist-related beliefs hold that work must be a way of personal fulfilment, but in reality, workers are alienated from work and exploited by the ownership classes of society. In the organizational belief system, work should help the individual to obtain a better status within the organization. According to the leisure ethic belief system, work has no meaning in itself and is only a means to produce goods and services, and earning money to buy them. Finally, the work ethic belief system sustains that work is good in itself and working is a way to be a useful member of society [72].

More recently, Bambra [32] described the value of work at three levels: work as economic relationship, work as self-identity, and work as social contract between society and the work. “Work as economic relationship” represents exchange of labor for money, which parallels the leisure ethic belief system of Buchholz [72]. “Work as self-identity” links to the humanistic belief system and involves workers as creative persons, attached to particular jobs, skills and location. Work provides additional rewards such as responsibility, achievement, pride and social support. Finally, “work as a social contract” considers work as a service to society and a mean of social integration and links to the organizational belief system [72].

*Work-related norms and values and LP of persons with chronic diseases* Schulz [73] found cross-country differences in the motivations of being a hard worker, which seem to align with Buchholz’s belief systems explained above [72]. Among American professionals, the economic aspect of work was most important (which aligns with the leisure work ethic system), among French and Norwegian professionals stimulating work tasks were (which aligns with the humanistic belief system). In India, economic factors, complying with expected social roles, and the desire to develop skills or to contribute something positive to society were motivations to engage in paid labor [71]. In Sweden a group of disabled workers were motivated to seek a job mainly because the need of being an efficient citizen, which links to the work ethic belief system but these workers identified work as an important part of their identity, which aligns with the humanistic belief system [74]. Moreover, employment commitment seems to vary in relation to the welfare regime. In a study comparing 18 European countries, people in Scandinavian welfare states have a higher employment commitment than people living in other welfare state types, even disadvantaged groups such as people with chronic diseases [75]. These findings suggest that cross-country differences exist in work-related norms and values, but some evidence for effects on the LP of persons with chronic diseases is only available [74, 75] for the work ethic belief system [72] and regarding work as a social contract [32]. Further, an interaction between the more generous and activating social-democrat system and higher work-commitment, also in persons with chronic diseases, was found [75].

#### Social Roles

Some values and norms are generally applicable, and others apply only to a selected group of actors [16, 24]. Social role refers to the conception of appropriate goals and activities for particular individuals or specified social positions [16]. In the context of LP among persons with a chronic disease, several roles might be of relevance. The “sick role” represents the normative expectations related to an ill person, such as legitimate withdrawal from social obligations, need of help and support to recover health. Further, a sick person must want to get well and must seek technically competent help. Persons with a chronic disease are expected to deal with their “normal” social roles (e.g. father, mother, worker), social expectations (e.g. productivity) and their sick role simultaneously [76]. Professional roles include norms that are supposed to lead professionals from the same occupation to have a similar behaviour and performance [77]. Professionalism comprises the ideals about the appropriate attitudes and actions of a person within a given profession [78]. This applies to WDP professionals. Finally, there is some evidence that gender roles influence LP [79].

*Social roles and LP of persons with chronic diseases* Due to the social expectations of their capabilities (the norms), the role-taking of persons with chronic diseases varies across countries. Normalization of the chronic illness can be the norm. In this case, the limitations associated with the illness are normalized to regain and maintain normal capacity, and the ideal role-taking of the person with a chronic disease is not giving in to illness [76]. Under these circumstances people might choose to hide their disability to show they are employable, and this might (unintentionally) be re-inforced by WDP professionals as they are trained to present their clients as dedicated and reliable employees in order to promote re-integration into work [80]. In these circumstances, social participation is thus put above exemption from normal social obligations [80]. In other countries, persons with chronic diseases are exempted from all social responsibilities. For example, Gartrell [70] demonstrated that in Cambodia, disabled people were regarded as inferior to healthy people, useless, and only capable of begging, and therefore, totally excluded from formal employment [38]. Professional norms might be supported by training but also by guidelines. Several countries have recognized the critical role of General Practioners (GPs) to reduce sick leave periods and stimulate return to work. To support GPs in this task, measures such as medical guidelines and clearer structural procedures for sick-listing practices have been implemented successfully [1]. Regarding gender roles, there is clear evidence for cross-country differences [79], also between high-income countries [81]. In a study sample comprising 12 countries, gender did not affect the LP of persons with chronic diseases, suggesting that gender norms might have a direct effect on their LP (countries with a positive gender norm have higher LP in general and thus also in persons with chronic diseases) [12, 82].

### Culture

While norms, values and role-expectations affect behavior via group pressure, culture exercises its influence more covertly. Persons only notice the influence of one’s culture on ones behaviour when confronted with another culture. For example, a person from Chili visits a GP in the Netherlands with symptoms of a disease and is advised rest. She might only then notice that back home, symptoms of a disease are commonly treated with medication. Several scholars have developed and empirically tested cultural dimensions to classify countries [83–86].

#### Culture Type

All theoretical sources included in our searches include dimensions related to power. Hofstede distinguishes different degrees of power distance, ranging from egalitarian to hierarchical cultures. In hierarchical cultures, less powerful members expect and accept that power is distributed unequally to a larger extent [83]. Schwartz distinguishes hierarchy and egalitarianism values. Hierarchical cultures legitimize an unequal distribution of power, roles and resources, which leads to power differences and hierarchical systems and this also leads to specific values such as social power, authority, humility and wealth [84], illustrating the interconnection between culture and norms. In egalitarian cultures, conversely, individuals recognize one another as moral equals who share basic interests as human beings, leading to societal values such as equality, social justice, freedom, responsibility and honesty [84]. Douglas has developed the “grid-group cultural analysis” which describes hierarchy an “up-down, down-up” culture. Douglas is more critical than Schwarz though and states that egalitarianism equals an inside-outside type of culture, that has strong barriers that identify and at the same time separate, the community from non-members [85].

The second dimension varies from a collectivism to individualism. In collectivist cultures, the interest of the group prevails over the individual interest. People who grow up in a more collectivist culture think of themselves as a part of a group, an “ingroup”. This ingroup is the major source of identity, and protection and therefore, one owes loyalty to the ingroup [83]. Schwartz describes collectivism in terms of the value conservatism. According to Schwartz, cultures in which conservatism is predominant, persons are viewed as embedded in the collective and find meaning in life largely through social relationships, through identifying and sharing way of life with the group. This gives rise to societal values such as social order, respect for tradition, family security and wisdom [84]. Douglas distinguishes the degree of integration of the individual with the group, ranging from minimal incorporation to the complete incorporation [85].

Individualist societies, according to Hofstede, regard the individual interest as most important. One is expected to look after himself or herself and his or her nuclear family, in which children grow up learning to think of themselves as an independent person [83]. In Schwartz’s theory, individualism is represented by the degree of autonomy, which refers to viewing persons as autonomous individuals who find meaning in their own uniqueness and seek to express their own internal attributes (preferences, traits, feelings and motives) and who are encouraged to do so [84]. For Douglas, an individualistic society would be the society where persons have maximum freedom to negotiate with each other, and where “effective group boundaries” and “constrains in private dealings” are lacking [85].

*Culture and LP of persons with chronic diseases* Although direct empirical evidence is lacking, both ends of the hierarchical-egalitarian dimension seem advantageous for the LP of persons with a chronic disease. Hierarchical cultures have been positively associated to work centrality and the view of work as a duty [84], and as such might be favourable for LP of people with chronic conditions. Egalitarian cultures are associated with the norm of work as a right [84], and might treat persons with chronic diseases more often as individuals with equal value for society and with the right to work.

Collectivist cultures might affect the LP of persons with chronic diseases in two contradictory ways. Collectivist cultures value conservatism, which is has been associated with the view of work as a duty or obligation to the group [84]. Thus, a collectivist society could exert social pressure on his members to work, which might lead to higher LP among the persons with a chronic disease. However, another characteristic of collectivist cultures is that they promote social support between members of a group (e.g. extended family, work colleagues or neighbours) [87], and thus the group might take responsibility for his members and this might decrease the social pressure to work in case of a chronic disease.

Individualistic cultures have been associated with the value of work centrality [88], which relates to the societal norm of work as a right [84]. Moreover, individualistic cultures might also promote being self-supportive and independent. Thus, this culture could favour LP of people with chronic conditions as well but for other reasons than collectivist cultures.

### Organization of WDP in Practice

As described in the introduction, the fourth basic factor of our model constitutes how practice is organized, including its professionals and organizations [16, 22]. With respect to the organizational structure of WDP in practice, some of the professionals involved are general practitioners (GPs), specialist doctors, professionals of vocational rehabilitation, professionals in the disability and insurance systems, social service professionals [6, 77, 78]. Generally, positive effects of the mere existence of a profession represented by shared professional norms, accreditation and professional training affected the quality of performance of medical professionals more that financial incentives [77].

There is vast support for the positive impact of the existence of organizations that support ALMPs, [3, 12, 57]. These are for example re-integration agencies, social insurance institutes and occupational health agencies.

*Organization of WDP in practice and LP of persons with chronic diseases* The professionals involved in the specific case of supporting LP of people with chronic diseases, as well as their training may vary across countries. Finally, employers might also be involved in promoting LP of persons with a chronic disease. Occupational Health and Safety (OHS) legislation in Europe defines the OHS tasks of employers, and employees with chronic diseases might profit from them. However, the implementation of this EU-Directive varies in practice, depending on the history of employer involvement and the quality of the Labour Inspectorate [89]. Moreover, larger formal employer responsibilities in return to work (RTW) guidance do not seem to guarantee more positive effects on the return to work of cancer survivors [90] and the labor participation of persons with chronic diseases in general [90].

### Labor Market Structure

The characteristics of the labor market demands and supplies constitute the fifth factor and differ across countries [91]. Most Western countries have now experienced labor market transitions from a predominant industrial production sector to the service sector [92]. Moreover, technological changes and globalisation have increased the need for skilled workers and made the labor market more competitive [93]. Several developments have increased non-standard types of work such as temporary or part-time work [1, 76].

The formal labor market includes regulations and labor market institutions to protect workers, which are absent in informal labor markets [94]. The informal economy plays a major role though in employment creation, income generation and production in many countries. Informal employment includes persons employed within informal sector enterprises, employees in informal jobs, contributing family workers and own-account workers that produce goods for use by their household [95]. The proportion of workers engaged in informal employment is particularly high in middle and low income countries [96].

The type of contract is characteristic for the labor market structure. In the EU, now 40% of the workers has a non-standard work contract [97]. The percentage of permanent jobs is decreasing and it is possible that permanent jobs concentrate in specific groups [98]. Many labor markets allow part-time employment, which can provide flexibility for workers and for employers. Self-employment represents 15% of total employment within the EU and is on the increase [97, 99]. Self-employed workers are the workers who work on their own account or in association with other partners [95]. This type of employment can provide more flexibility than conventional types of work [99, 100].

At the supply side, labor population characteristics can reflect the opportunity that a job seeker with certain characteristics can effectively find a job [95] and thus is a competitor to a persons with chronic diseases. The labor population can be characterized first by national labor force participation rate (LFPR), the relative size of the supply of labor available. Second, the employment-to-population ratio is the proportion of the working-age population employed in a given country, reflecting the ability to create employment within an economy. Thirdly, unemployment rate represents the proportion of people that does not have a job and is actively looking for work [101].

*Labor market characteristics and LP of persons with chronic diseases* Informal work is often associated with job insecurity and precarious work conditions [96]. Sustaining labor under these circumstances may be more difficult and detrimental to persons with chronic diseases thus, this group may abstain to engage in this type of work. However, if an important share of a country’s employment is available in this sector, persons with chronic diseases may have lower LP or unsafe working conditions.

Temporary contracts might be disadvantageous for persons with a chronic health condition, because they might have more difficulties to re-enter the labor market when they compete with many healthy unemployed. Part-time work can allow persons with chronic diseases to participate in the labor market [1, 67, 102, 103]. Part-time jobs can facilitate workers coping with fatigue and other symptoms that are often associated with chronic conditions [67]. In fact, persons with chronic diseases are more likely enrolled in part-time jobs than fulltime jobs [67, 68].

Self-employment can also provide flexibility for individuals with chronic conditions to accommodate their health condition with work [99, 100]. Persons with a work-limiting condition are more likely to be self-employed, particularly when they are male [100] or living in Southern European countries such as Greece and Portugal [99]. It might provide equal or higher levels of work satisfaction among people with a disability compared to the healthy population [90].

High unemployment, such as during an economic recession, might increase the competition between the healthy and unhealthy working population, and appeared to be disadvantageous for persons with mental health problems in a study across 27 European countries [104]. High unemployment might thus be disadvantageous for the LP of persons with chronic diseases.

### Transferability and the CCC Model for LP: the LOLA-tool

The academic groups that tested the model expressed a need for a simple tool for a first and global estimation of the transferability of a law, policy or intervention developed in the context of one country, to one’s own country. This need was noticed among students ranging from bachelor to post-graduate level as well among researchers and professionals attending conferences. To address this, we simplified the five main factors into four questions (norms and values were combined with organization in practice): addressing the similarity of Legislation, Organizations and the professional norms and values, Labor market characteristics and the degree of Amazement about the law, policy or intervention (as cultural differences reveal themselves by taking one by surprise [83]) (LOLA, see Table 2). The LOLA-tool was introduced at the EUMASS conference in 2018, next presented at ten occasions and received well.

**Table 2:** LOLA-tool for assessing transferability of X (law, policy or intervention in relation to LP of persons with chronic diseases) from one country to another country

Question	Rating between 0 (No, not at all) to 10 (Yes, completely)
Is the Legislation similar?	
2. Are the Organizations and professional views in relation to X similar?	
3. Are the Labour market characteristics similar?	
4. Am I not Amazed at all by X? (When assessing transferability to one’s own country) Will the people in country Y not be Amazed at all? (When assessing transferability to country Y)	
Estimate of transferability	Σ = (0 = not at all……40 = definitely transferable)

## Discussion

In the context of WDP, this paper presents the CCC model for LP of persons with chronic diseases, based on theory (NIT), empirical evidence and expert feedback. The model is visually represented by a succinct 5-factor model (Fig. 2), and a comprehensive model representing the subfactors (Fig. 3). The model focuses on institutions related to LP in the context of chronic illness at the different layers of a society, and adds two factors: the organization of WDP in practice and labor market characteristics.

Researchers can use the model for qualitative comparative (multimethod) case studies, in order to better understand the country’s systems, similar to the use of NIT by Kümpers and colleagues when analysing cross-country differences in health policy for elderly [18]. Interviews, documentary research, quantitative data, and national as well as international registries can be used for such case studies. The model might also be used for quantitative studies that aim to explain differences in LP rates of persons with chronic diseases by multiple determinants, if LP measures are comparable between countries [10]. Up until now and as far as we know, no model for cross-country comparison in the context of WDP, nor for LP among persons with chronic diseases in particular, is available. A model for cross-country comparison of mental health care systems (WHO-AIMS) includes six domains of which five overlap with four of our factors (i.e. legislation, norms and values, culture, organization): (1) policy and legislative framework that overlaps with legislation; (2) education of the public at large, that overlaps with norms and values and with culture; (3) mental health services, (4) mental health in primary care and (5) human resources, that overlap with organization. The sixth domain of WHO-AIMS, (6) monitoring and research, does not align with one of our five main factors while our factor ‘labour market characteristics’ was not represented in WHO-AIMS [105].

The CCC model can also be applied in academic education. Academic teachers in the fields of e.g. public health, occupational health, law, medicine, health and social sciences can use the model to teach students about the complexities of cross-country comparisons of WDP law, policies and interventions. Our experiences in 28 courses ranging from bachelor to post-graduate level, including participants from all continents, showed the model to be instructional.

### Reflection on the Model

We would like to warn that the CCC model for LP is not normative with regard to the question whether LP is always better for persons with a chronic disease or disability. Labor participation might put too high a burden on the individual’s physical and mental wellbeing, particularly in the context of severe illness, low job quality, insecurity of income due to being self-employed or having part-time work [76], and private demands such as care for children and parents. Quality of life or the experience of successfulness of LP [106] were not taken into account when developing this model. Our second warning is that this sociological model focuses on understanding and not on causal explanations. Causal relationships need to be tested in quantitative research, similar to the study on determinants of public health policy success across 43 European countries [107].

### Methodological Considerations

The model’s advantage is its strong theoretical basis, which is the first of six quality criteria for cross-country comparisons in the context of health system and policy research [108]. NIT is a well-known and widely studied theory in the fields of economics, political sciences and sociology. Further, the model builds on empirical evidence and includes inputs of researchers in the field of LP and cross-country comparative research. Although studies were biased towards Europe, results from global studies (e.g. by International Labour Organization, Organisation for Economic Co-operation and Development, World Bank, and World Health Organisation) were included as well as studies on beliefs and culture from the Middle East and Asia [69–71, 79, 83, 85–87].

However, the search for empirical evidence for the relationships between subfactors and LP of persons with a chronic disease was not completely systematic and did not include an assessment of the quality of the empirical studies. Moreover, because of a lack of empirical studies, evidence for some subfactors was general and not specific for LP and/or persons with a chronic disease.

### Recommendations for Further Research

Research on cultural differences between countries is needed to further validate the model. The CCC model for LP can support the much needed cross-country comparative research with case studies, aiming to learn from each other [107]. The 5-factor CCC model (Fig. 2) can support a systematic discussion of possible explanations for the cross-country differences and similarities found in quantitative comparisons and systematic reviews. Further, the generic theoretical background allows for applying to slightly different topics as the cross-country comparison of Occupational Health Physiotherapy practice in Australia and Japan [109] or, with some adaptation be applied to other target groups such as the older workers or workers with mental disorders.

### Recommendations for Practice

Regarding practice, the simple LOLA-tool (Table 2) supports policy-makers and practitioners to estimate cross-country transferability of a law, policy or intervention in relation to labor participation of persons with chronic diseases.

### Conclusion

On the basis of theory, empirical evidence and expert knowledge, the CCC model for LP of persons with chronic diseases was developed (Figs. 2, 3). This model supports systematic cross-country comparisons with case studies with the aim of policy learning, estimating the transferability of laws, policies or interventions from one country to another, and to systematically interpret country differences found in quantitative studies.

## Data Availability

The data generated in the literature review are included in this article; data on the development of the model since its first concept are available from the first author upon reasonable request.

## Code Availability

Code availability does not apply to this research.
